# Personalized Neoantigen-Pulsed DC Vaccines: Advances in Clinical Applications

**DOI:** 10.3389/fonc.2021.701777

**Published:** 2021-07-26

**Authors:** Lin Tang, Rui Zhang, Xiaoyu Zhang, Li Yang

**Affiliations:** State Key Laboratory of Biotherapy and Cancer Center, West China Hospital, Sichuan University, and Collaborative Innovation Center for Biotherapy, Chengdu, China

**Keywords:** personalized neoantigen, DC vaccine, tumor, immunotherapy, clinical applications

## Abstract

In the past few decades, great progress has been made in the clinical application of dendritic cell (DC) vaccines loaded with personalized neoantigens. Personalized neoantigens are antigens arising from somatic mutations in cancers, with specificity to each patient. DC vaccines work based on the fundamental characteristics of DCs, which are professional antigen-presenting cells (APCs), responsible for the uptake, processing, and presentation of antigens to T cells to activate immune responses. Neoantigens can exert their antitumor effects only after they are taken up by APCs and presented to T cells. In recent years, neoantigen-based personalized tumor therapeutic vaccines have proven to be safe, immunogenic and feasible treatment strategies in patients with melanoma and glioblastoma that provide new hope in the treatment of cancer patients and a new approach to cure cancer. In addition, according to ClinicalTrials.gov, hundreds of registered DC vaccine trials are either completed or ongoing worldwide, of which 9 are in early phase I, 191 in phase I, 166 in phase II and 8 in phase III. Hundreds of clinical studies on therapeutic tumor vaccines globally have proven that DC vaccines are stable, reliable and very safe. However, in this process, many other factors still limit the effectiveness of the vaccine. This review will focus on the current research progress on personalized neoantigen-pulsed DC vaccines, their limitations and future research directions of DC vaccines loaded with neoantigens. This review aims to provide a better understanding of DCs biology and manipulation of activated DCs for DCs researchers to produce the next generation of highly efficient cancer vaccines for patients.

## Introduction

Malignant tumors are still an acute threat for people worldwide and the incidence and mortality from cancer are still rapidly growing. GLOBOCAN showed an estimated 19.3 million new cases and 10 million cancer deaths worldwide in 2020; at the same time, an estimated 28.4 million new cancer cases are projected to occur in 2040 (1). Therefore, it is still difficult to find proper and effective ways to fight cancer.

After decades of effort, conventional methods and systems for treating cancer have been developed, including surgery, radiotherapy and chemotherapy alone or in combination. Surgery is the preferred treatment for most tumors; however, it is a traumatic and local treatment that easily leads to surgical complications. Although radiotherapy is the most suitable method for tumors in all parts of the body, the radiation dose that the body can withstand is limited, and normal cells are also damaged when tumor cells are destroyed. Although chemotherapy is successful for some tumors, such as testicular tumors, it can cause severe side effects, such as hair loss, anemia and organ damage, reducing the patients’ quality of life (2, 3). Because of the side effects of conventional treatments, cancer immunotherapy has been developed as a therapeutic method with better tumor targeting, safety, and a lower toxicity.

**Figure 1:**
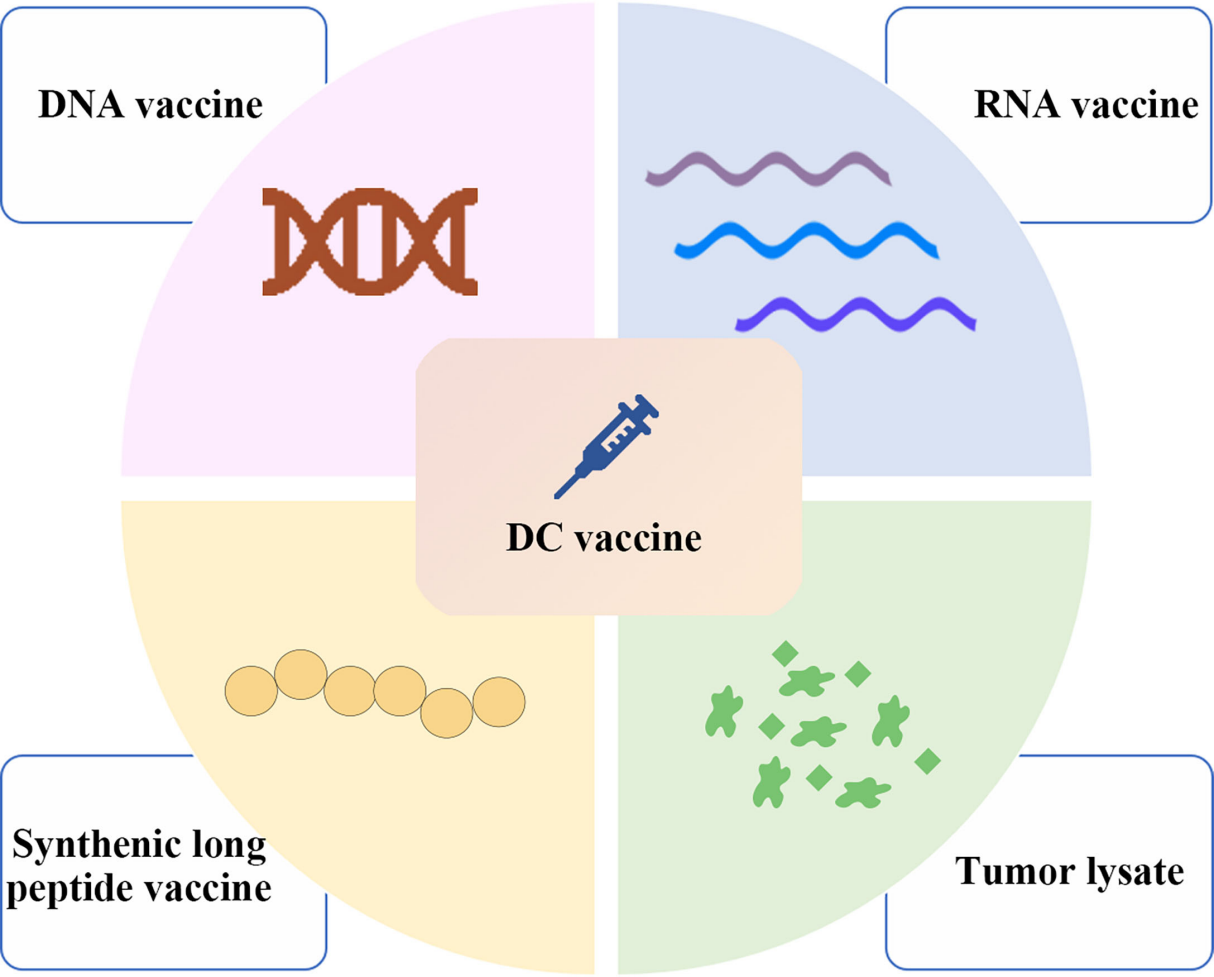
Major types of neoantigen vaccines in clinical research. Neoantigen vaccines mainly include nucleic acid vaccines consisting of DNA and RNA vaccines, synthetic long peptide vaccines and tumor lysate vaccines. In addition to being used separately as vaccines, these neoantigens formulations can also be loaded onto DCs. Therefore, when working with DC vaccines, still the choice needs to be made between loading with either peptides, RNA, DNA or tumor lysate.

**Figure 2:**
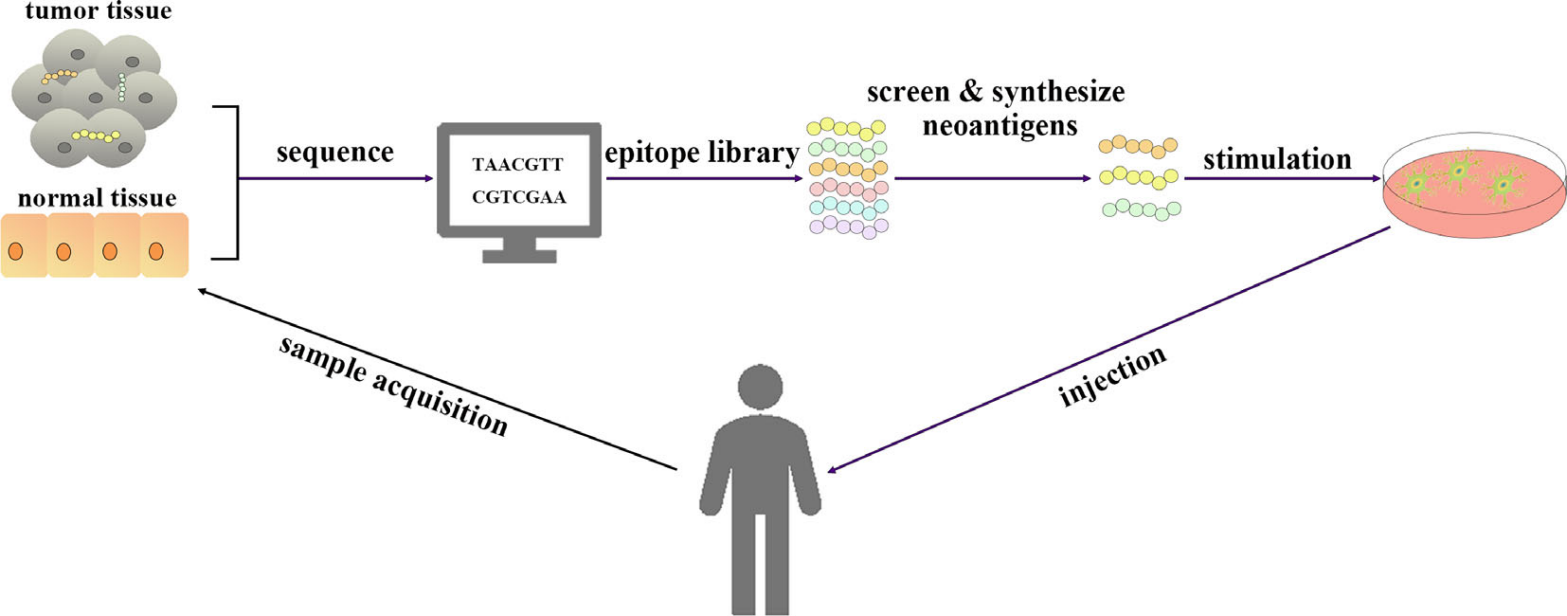
Schematic diagram of personalized neoantigen-pulsed DC vaccines. Tumor tissue and normal tissue of patients were sequenced. The epitope library is processed by bioinformatics methods, from which immunogenic neoantigens are screened and synthesized. The DC vaccines loaded with neoantigens are prepared by using DCs extracted from the peripheral blood of patients and injected into patients.

Cancer immunotherapy relies on the individual’s own immune system to recognize and control cancer progression to fight and cure cancer (4). At the same time, cancer immunotherapy has been developed to enhance the antitumor response of the immune system and reduce off-target effects and other serious side effects of other conventional therapies (5). There are five main types of cancer immunotherapy (6):

(i) Immune checkpoint inhibitors, in which the most extensive strategies involve the use of programmed death 1/programmed death ligand 1 blockade (PD1/PD-L1 blockade) and cytotoxic T lymphocyte-associated antigen-4 inhibition (CTLA-4 inhibition). Immune checkpoints are immunosuppressive pathways that regulate the immune response to maintain tolerance and protect the surrounding tissues. This property is used by tumor cells to escape the attack of immune cells, and immune checkpoint inhibitors can inhibit immune checkpoint activity and reactivate the immune response of T cells to the tumor to achieve an antitumor effect (7).
(ii) Cytokines, which contain three main types (interleukins, interferons, and granulocyte–macrophage colony-stimulating factor (GM-CSF)) (8), are the first class of approved immunotherapies for clinical use and have effects *via* stimulating immune cells directly (6, 9).
(iii) T cells and natural killer (NK) cells, in which T cells include engineered T cells and non-engineered T cells such as adoptive tumor infiltrating lymphocyte (TIL) and cultivated T cells. Engineered T cells contain chimeric antigen receptor T cells (CAR-T) and T cell receptor T cells (TCR-T), and CAR-T cells can trigger the death of tumor cells by recognizing the targeted antigens on tumor cells (10), and the antitumor activity of TCR-T cells is mainly stimulated by tumor-associated antigens presented by major histocompatibility complexes (MHCs) (11). NK cells also include engineered NK cells such as CAR-NK cells and many trials are under way.
(iv) Agonistic antibodies, which can specifically bind to receptors on the surface of T cells, triggering intracellular signaling pathways and inducing T cells to function as effectors to kill tumor cells (12).
(v) Cancer vaccines include those based on tumor cell lysates, nucleic acids, and peptides, which contain or can encode neoantigens (13). Neoantigen vaccines are an attractive type of cancer vaccine. In addition to being used separately as vaccines, DNA, RNA, peptide and tumor lysate can also be loaded onto DCs (**Figure 1**). Although DNA can be easily manipulated by molecular engineering, the successful use of the first generation of drug delivery platforms in humans is limited, and they tend to rely more on electroporation and it is also limited by its potential to integrate into the genome (14). On the other hand, there is no potential risk for RNA to integrate into the genome; however, it is still affected by RNase degradation although modification may prolong its half-life (14). In addition, synthetic long peptide (SLP) is easy to store, low toxicity and appropriate adjuvants are required (14). Therefore, when working with DC vaccines, the choice needs to be made between either loading with peptides, RNA, DNA or tumor lysate. Furthermore, DCs pulsed with neoantigens *ex vivo* to treat patients can effectively induce anti-tumor immune responses induced by activated T cells (13). Hundreds of research and clinical trials have been conducted or are underway since the first DC vaccine, sipuleucel-T, was approved for clinical use in 2010 (15). Although the safety of DC vaccines has been demonstrated in several clinical trials, several clinical trials have still failed due to the lack of clear efficacy (16, 17). The emergence of personalized neoantigens that were isolated, identified and selected from the patients’ tumors and their entry into the body after loading on DCs *ex vivo* can promote the efficient presentation of neoantigens by DCs to T cells to exert an anti-tumor role (16) (**Figure 2**). In this review, we summarize the progress and clinical application of personalized neoantigen-pulsed DC cancer vaccines.

## Personalized Neoantigens

Neoantigens are a series of peptides with tumor specificity that are present in proliferating tumor cells but not in normal tissues. Therefore, they are different from tumor-associated antigens (TAAs) mostly present in both normal and tumor tissues, which also include viral antigens (18, 19). At the same time, they are derived from viral proteins such as open reading frame-derived epitopes in the viral genome and tumor somatic nonsynonymous genetic alterations, including genomic variant level such as single nucleotide variants (SNVs), insertion-deletions, gene fusion, frame shift mutation and transcriptomic and proteomic variants (20–23). The change in peptide sequence and its spatial structure can result in a stronger affinity for major histocompatibility complexes (MHCs), and therefore, making it more likely to be recognized by T cells to induce antitumor immune responses (24). In general, neoantigens are divided into two subgroups: shared neoantigens and personalized neoantigens (25). Shared neoantigens are common in some tumor types and can be used broadly to treat patients who have the same tumor type and express these antigens; however, there are antigenic differences between different patients and different tumors, limiting the role of shared antigens (26, 27). Unlike shared neoantigens, personalized neoantigens are a class of antigens specific to individual patients and tumors. Since tumors of the same cancer type can vary greatly, personalized treatment with personalized neoantigens is a better way to ensure a response by each cancer type (25).

A series of completed or ongoing clinical trials on personalized tumor neoantigens is listed in **Table 1** according to the data on ClinicalTrials.gov. In the trial conducted by Ott (28), they enrolled 10 patients, 8 of whom displayed a high degree of melanoma-related mutations as expected, and then 13-20 immunizing long peptides were synthesized for each patient. Finally, 6 patients completed the full series vaccinations. No disease recurrence was observed in 4 patients during a median follow-up period of 25 months (range 20-32 months) after vaccination. Two other patients suffered disease recurrence but also had a complete response after anti-PD1 therapy, which still shows that personalized neoantigen-based vaccines are safe and feasible and could be used in the clinic.

**Table 1:** The clinical application of personalized neoantigen vaccines.

Tumor types	Phase	Status	Participants	NCT number
Melanoma	Phase I	Completed	20	NCT01970358
Pancreatic Cancer	Phase I	Recruiting	60	NCT03558945
Kidney Cancer	Phase I	Recruiting	19	NCT02950766
Bladder Cancer	Phase I	Recruiting	15	NCT03359239
Pancreatic Cancer	Phase I	Recruiting	20	NCT04161755
Non-Small-Cell Lung cancer	Phase I	Recruiting	20	NCT04487093
Glioblastoma	Phase I	Recruiting	56	NCT02287428
Melanoma	Phase I/II	Recruiting	25	NCT03715985
Bladder Cancer				
Non-Small-Cell Lung cancer				
Melanoma	Phase I	Recruiting	30	NCT04072900
Breast Cancer	Phase II	Recruiting	70	NCT03606967
Small Cell Lung Cancer	Phase II	Not yet recruiting	27	NCT04397003
Diffuse Intrinsic Pontine Glioma	Phase I	Not yet recruiting	30	NCT04749641
Melanoma	Phase II	Active, not recruiting	60	NCT02129075

### Identification of Personalized Neoantigens

The identification of personalized neoantigens is an important part of tumor immunity therapy to allow personalized neoantigens to have an effect in each patient. The common approach is to compare DNA sequences in tumor tissues with those in normal tissues using high-throughput sequencing technologies (next-generation sequencing, NGS), which is rapid and efficient (29). However, many of the detected DNA mutations are not expressed as they are noncoding mutations or nonsense mutations, which poses new challenges to identify neoantigens (29). With the progress of sequencing technology, a more efficient and feasible sequencing technology with a lower false-negative rate was born: whole-exome sequencing technology (the exome is the protein-encoding part of the genome), which is currently widely used to identify personalized neoantigens (30). The mutant amino acid sequence that can be expressed needs to be translated and processed into short peptide fragments. These also need to be expressed on the cell surface in complex with MHC molecules to be recognized successfully by the immune system (19). Therefore, there are several crucial factors that determine whether a mutation can produce an effective personalized neoantigen: (i) whether the mutated DNA sequence can eventually be expressed and processed into short peptide fragments at the protein level; (ii) the ability of peptides to be presented and their affinity to MHC molecules; (iii) the affinity of the complex formed by the mutant peptides and MHC to TCR (31).

Because of lots of work involved in comparing high-throughput sequencing data, the development of computer simulation experiments or tools has effectively promoted the identification of personalized tumor neoantigens. On the one hand, for different neoantigen sources, there are corresponding computational tools. For single nucleotide variants (SNVs), small insertions and deletions (INDELs), or gene fusion at the genomic variant level, pVAC-Seq, TSNAD, CloudNeo, Tlminer, MuPeXI, Neopepsee, and INTEGRATE-Neo are usually utilized (32–37). For alternative transcript splicing at the transcriptomic variant level, NeoantigenR is widely used (38). On the other hand, these mutations need to be ranked according to their affinity with individual autologous MHC molecules; for this, the tools NetMHC, SMMPMBEC and SMM, among others, are used (39–41). Of the MHC molecule, MHC-I is directly related to neoantigen presentation on tumor cells, and the methods of using the MHC-I molecule to predict neoantigens are relatively mature at present, while CD4^+^ T cells recognize predicted neoantigens presented by MHC-II molecules. Compared to MHC-I molecules, where the peptide-binding groove is closed at both ends, the binding groove of MHC-II molecules is open at both ends and can deliver longer peptides (11-20 amino acids) (42). However, there is currently a lack of robust and rich databases and effective tools for assessing the interactions between MHC-II molecules and peptides compared with what is available for MHC-I (42). The further development of bioinformatics resources and the use of other cross-disciplinary methods are expected to improve neoantigen identification.

### Personalized Neoantigens Manufacturing

Personalized neoantigens are a unique class of neoantigens specifically prepared for each patient; therefore, a rapid, simple, and mature system for the synthesis of personalized neoantigens is needed, as this is the first step of manufacturing neoantigens (43). In addition, the formulation of neoantigens such as buffering agents and surfactants are another crucial factor due to the different compositions and properties of each personalized neoantigen and these other components play important roles in ensuring the solubility and stability of neoantigens (44). The next step is purification. Many systems are used to purify neoantigens such as RP-HPLC and flash-like systems. With the development of new technologies, an increasing number of manual operations have been replaced by automated processes such as auto-sampling systems and ultra-performance liquid chromatography (UPLC) (45), saving time while enhancing the productivity and quality. The last step is lyophilization to make the newly prepared neoantigens easier to transport and store until used. Similarly, as technology advances, the processing of personalized neoantigens will become faster and more efficient, saving the patient precious time and increasing the effectiveness of cures.

## Personalized Neoantigens-Pulsed DC Vaccines

### Definition and Types of DCs

Immune cells, which include B cells, T cells, natural killer cells (NK cells) derived from lymphoid stem cells and neutrophils, eosinophils, basophils, and monocytes derived from myeloid progenitor, produce an immune response to resist the invasion of bacteria and viruses, kill tumor cells and maintain the human body’s immune balance. DCs, often differentiated from monocytes, are professional antigen-presenting cells and are responsible for efficient uptake, processing and presentation of antigens, which can not only teach naive T cells to become antigen-specific cytotoxic T cells (CTLs) through antigen presentation but also allow for interaction with other immune cells in the body, such as NK cells, T cells and B cells, activating the immune system to recognize and kill tumors (46).

In humans, committed DC precursors (CDPs) in bone marrow are divided into two major subsets of DCs, plasmacytoid DCs (pDCs) and conventional DCs (cDCs), which include two major categories-cDC1 and cDC2 based on their phenotype (47). These cells circulate in the blood and continue to enter the lymphoid organs and peripheral tissues as a supplement to DCs. For pDCs, surface markers mainly include CD123, CD303, CD304, and CD45RA, and they specifically secrete type I interferons (IFN-I) while presenting antigens to T cells and activating T cells (48, 49). For cDC1, surface markers mainly include Cleca9A, XCR1, and CD141, and cDC1 have the ability to cross-present and induce cytotoxic T cell immune responses and can also significantly stimulate the immune response of allogeneic or autologous CD4^+^ T cells (48, 49). For cDC2, surface markers mainly include CD1c, CD1a, and CD103, which can present soluble antigens but rarely present antigens derived from necrotic cells (48–50). In conclusion, different DCs play different physiological functions in the body, promoting the important role of DCs in immune regulation.

### DC Vaccines

The principle of preparing a DC vaccine is simple. The precursor cells of DCs in patients are isolated and cultured *in vitro*, loaded with tumor antigens, and then transferred back into patients. Then, the antitumor effect can be exerted by specific antitumor T cells stimulated by DCs. After nearly 10 years of effort in the field of DC vaccines, in 2000, DC-based immunotherapy was used for the first time in a patient with a primary intracranial tumor. The patient received 3 treatments with an allogeneic MHC class I glioblastoma peptide-pulsed DC vaccine. The trial showed that the DC vaccine was tolerated, and the patient received a positive immune response. However, no objective clinical response was observed (51). In 2010, the United States FDA approved Sipuleucel-T as the first therapeutic DC vaccine for prostate cancer. Sipuleucel-T consists of peripheral blood mononuclear cells (PBMCs), which include APCs, activated *ex vivo* by PA2024, a recombinant protein including mainly prostate-specific antigen and prostatic acid phosphatase (15).

Philip W. Kantoff’s group divided 512 patients into two groups at a ratio of 2:1 to receive treatment with Sipuleucel-T and placebo every two weeks by intravenous injection, for 3 treatments in total (52). The results showed that the 36-month survival was 31.7% in the Sipuleucel-T group and 23% in the placebo group. The median survival duration in the Sipuleucel-T group was 25.8 months, an increase of 4.1 months compared with 21.7 months in the placebo group. This revealed that the drug could significantly prolong the survival period, suggesting that the DC vaccine can give patients a survival benefit. Another clinical trial on glioblastoma also showed superior efficacy of a DC vaccine. ICT-107 is an autologous DC vaccine pulsed with 6 different peptides targeting glioblastoma. In a prior phase I study, 21 patients with glioblastoma administered ICT-107 showed good tolerance and in 16 newly diagnosed patients, 6 patients did not show tumor recurrence, which showed that this DC vaccine was well tolerated and possessed antitumor activity (53).

In the following phase IIb trial conducted by Patrick Y. Wen, among HLA-A2^+^ patients with a matriculated MGMT promoter, progression-free survival (PFS) in the ICT-107 group (24.1 months) was significantly higher than that in the control group (8.5 months) and the patients in the ICT-107 group showed improved immune responses (54). Although many trials have focused on DC vaccines in recent years (**Table 2**), the basis of DC vaccines is the selection of immunogenic antigens to activate the immune system effectively in addition to the maturation of DCs. Because the antigens in each patient’s tumor are highly specific, DCs loaded with personalized neoantigens for fusion into therapeutic tumor vaccines are another attractive strategy.

**Table 2:** The clinical application of DC vaccines.

Tumor types	Phase	Status	Participants	Source of DCs	NCT number
Breast Cancer	Phase I/II	Completed	10	/	NCT02018458
Breast Cancer	Phase I/II	Completed	44	/	NCT01042535
Unspecified Adult Solid Tumor					
Breast Cancer	Phase I	Completed	31	monocytes	NCT00978913
Malignant Melanoma					
Colorectal Cancer	Phase I	Completed	6	monocytes	NCT01671592
Lung Cancer	Phase II	Completed	32	white blood cells	NCT00103116
Prostate Cancer	Phase II	Completed	13	/	NCT00970203
Hematological Malignancies	Phase I/II	Completed	10	/	NCT02528682
Gastric Cancer	Phase I/II	Recruiting	45	/	NCT04567069
Colorectal Cancer	Phase I	Recruiting	12	/	NCT03730948
Glioblastoma	Phase II/III	Recruiting	60	/	NCT03548571
Breast Cancer	Phase I	Active, not recruiting	15	/	NCT02063724

### Clinical Trial Progress of Personalized Neoantigen-Pulsed DC Vaccines

Tumor vaccines that rely on neoantigens alone cannot completely eliminate malignant tumors (55). The reason for this is not the neoantigen itself but more because most of the trials used neoantigens to solve the problem of the weak antigenicity of tumor cells but did not solve the problem of immune cell functional defects in cancer patients. Patients with malignant tumors usually have a low level of immune function, and it is difficult to initiate the antitumor immune response *in vivo*. One of the main reasons is that the function of antigen-presenting cells in patients is inhibited, and antigen-activated T cells cannot be effectively presented. Therefore, to achieve good clinical efficacy, immunotherapy should not only solve the problems related to antigens but also the problems of immunosuppression in tumor patients. In other words, when many tumor-specific antigens are injected into the body, it is necessary to ensure that they are efficiently taken up and presented by the body’s antigen-presenting cells and that a sufficient number of effector T cells are activated.

In 2015, the first personalized neoantigen-loaded DC vaccine began testing in a phase I clinical trial (56). They enrolled 3 melanoma patients with stage III resected cutaneous melanoma and treated them with ipilimumab. Then, they identified somatic mutations from their own surgically excised tumors by whole-exome sequencing and computer-simulated epitope prediction to screen for suitable neoantigens. Furthermore, 7 neoantigens selected from each patient were loaded with DCs isolated from PBMCs, cultured *ex vivo*, and then intravenously injected into the patients for a total of three treatments. After the treatments, an enhanced immune response triggered by T cells was observed, while three patients were all surviving and no autoimmune adverse reactions were observed, which showed that DC vaccines pulsed with personalized neoantigens was safe and reliable.

In another trial conducted by Ding’s group in 2020, they demonstrated for the first time the activity of a personalized neoantigen-pulsed DC vaccine in patients with advanced NSCLC (57). In their study, they enrolled 12 patients with advanced lung cancer and 13-30 peptide-based personalized neoantigens were isolated and identified from each patient’s tumor tissue. At the same time, PBMCs were derived from each patient, DCs were separated, then DCs were pulsed with the corresponding selected neoantigens to form a personalized neoantigen-pulsed DC vaccine to treat patients. Their study showed a 25% objective response rate, while a 75% disease control rate was observed after treatment of a personalized neoantigen-pulsed DC vaccine. In addition, only low-grade and transient side effects were observed, which also demonstrated that the vaccine was safe and able to induce specific T cell immune response. In particular, a patient with metastatic lung cancer whose main metastases were in bone, pelvis, and inferior vena cava lymph nodes failed to show a tumor response after three treatments. Then, he received personalized neoantigen-pulsed DC vaccine treatment, and after 5 doses of this vaccine, almost no metastatic lymph nodes and shrinking pelvic lesions were observed. There was a 29% reduction in overall tumor lesions, which showed a good therapeutic effect of the vaccine.

In Sarivalasis’s paper published in 2019, they present another phase I/II trial that uses personalized peptides, including tumor-specific neoantigens and TAAs derived from patients, and pulses them into DCs isolated from autologous monocytes (58). They will acquire the tumor specimens from each patient for NGS analysis. Then they will analyze the data to generate personalized databases, and up to 10 will be selected per patient by verifying the immune response of the candidate peptides to T cells isolated from the patient. This trial will investigate the feasibility and safety of a personalized neoantigen-loaded DC vaccine in patients with ovarian cancer and evaluate overall survival (OS) progression time and disease-free survival at 12, 24, and 36 months. This trial is the first of its kind to test a personalized neoantigen-pulsed DC vaccine in ovarian cancer patients. We look forward to its expected efficacy in a clinical trial, providing additional strong evidence of the efficacy and safety of a personalized neoantigen-pulsed DC vaccine and bringing benefits to patients.

In addition, according to ClinicalTrials.gov, there are several clinical trials around personalized neoantigen-loaded DC vaccines under way that are in phase I (**Table 3**). Although these trials are underway, the fact that they have been carried out only in the last decade shows that they are still forward-looking and innovative.

**Table 3:** The clinical application of personalized neoantigen-pulsed DC vaccines.

Tumor types	Phase	Status	Participants	Source of DCs	NCT number
Breast Cancer	Phase I	Completed	9	monocytes	NCT04879888
Triple Negative Breast Cancer	Phase I	Recruiting	5	/	NCT04105582
Gastric Cancer	Phase I	Recruiting	80	/	NCT04147078
Hepatocellular Carcinoma					
Non-Small-Cell Lung Cancer					
Colon Rectal Cancer					
Pancreatic Adenocarcinoma	Phase Ib	Recruiting	12	PBMC	NCT04627246
Non-Small-Cell Lung Cancer	Phase I	Recruiting	6	monocytes	NCT04078269
Advanced Biliary Tract Tumor	Phase I/II	Recruiting	40	/	NCT02632019
Non-Small-Cell Lung cancer	Phase I	unknown	20	/	NCT02956551
Liver Cancer	Phase I	unknown	24	/	NCT03674073
Non-Small-Cell Lung cancer	Phase I	unknown	30	/	NCT03871205
Glioblastoma	Phase I	Enrolling by invitation	10	/	NCT03914768
Colorectal Cancer	Phase I/II	Active, not recruiting	25	/	NCT01885702
Non-Small-Cell Lung cancer	Phase I/II	Not yet recruiting	20	peripheral blood	NCT03205930

## Personalized Neoantigen-Pulsed DC Vaccines in Combination With Other Therapies

The combination of personalized neoantigen-pulsed DC vaccines with other strategies, such as chemotherapy and immune checkpoint inhibitors, is another attractive approach to enhance the tumor therapeutic vaccine efficacy. Chemotherapy is considered an immunotherapy partner to improve immunotherapy efficacy by enhancing antigen production and presentation, and inducing T cell immune response, although it still has several side effects (59). In a trial conducted by Batich and colleagues (60), cytomegalovirus antigen pp65 was found to be present in glioma cells instead of surrounding normal tissues. Then, they used a pp65-pulsed DC vaccine combined with dose-intensified temozolomide, which is a chemotherapeutic drug to treat glioma. As expected, the median PFS was 25.3 months and OS was 41.1 months, and both were much higher than the statistical median survival of patients (less than 15 months) with newly diagnosed glioma.

Immune checkpoints exert strong immunosuppressive effects to block the antitumor immune response; thus, neoantigen vaccines combined with immune checkpoint inhibitors, which involve mainly specific monoclonal antibodies such as anti-PD-1, anti-PD-L1, and anti-CTLA-4 antibodies, are thought to generate strong a T cell immune response to kill tumors (7, 61, 62). In Sahin’s trial, neoantigen-specific T cells were PD1^+^ and after the neoantigen vaccine, PD-L1 upregulation was observed (63). Then, anti-PD1 treatment was applied after the neoantigen vaccine, and a complete response to the neoantigen vaccine was observed. In a phase I trial, the combination of MART-1 peptide-pulsed DCs and tremelimumab, an anti-CTLA-4 antibody, was used for 16 patients with melanoma and they acquired a higher durable objective tumor response rate than treatment alone (64). In addition, a trial conducted by Ding showed that an enlarged tumor was still observed in a patient with lung cancer after treatment with a personalized neoantigen-pulsed DC vaccine (57). When nivolumab, an anti-PD-1 antibody, was combined with this DC vaccine, the patient’s tumor became cavitated, which demonstrated the superiority of combination therapy for cancer.

## Factors That Limit the Effectiveness of Personalized Neoantigen-Pulsed DC Vaccines

Although the current clinical application shows efficacy, personalized tumor neoantigen-pulsed DC vaccines are still limited in several aspects. (i) The selection of neoantigens: The sequencing and screening of tumor neoantigens requires individual detection and analysis for each patient’s tumor, which is a complex and time-consuming process. Additionally, the manufacture of neoantigens requires a better manufacturing conditions to ensure the consistency of neoantigens (65). As a result, the development and wide application of advanced technology are urgently needed. It is believed that the time and production cost of this process will be greatly reduced in the near future. (ii) The source and maturation conditions of DCs: DCs applied in personalized neoantigen-pulsed DC vaccines are also individualized, and it is necessary to extract DCs from each patient for separate culture. In addition, mature DCs are needed to enhance antigen processing, presentation and stimulate B and T cells. Antigens, cytokines such as GM-CSF and other factors such as LPS could stimulate DCs maturation. This process still has problems such as the intensive labor required for the *ex vivo* culture process and the skill required for inducing DC maturation. Thus, in future studies, efforts are needed to optimize *ex vivo* culture while inducing mature and high-quality DCs (66–69). (iii) The efficiency of DC migration: DCs injected back into patients should migrate to the lymphoid organs to stimulate T cells to achieve effective immune responses, and some proinflammatory cytokines, such as prostaglandin E_2_ (PGE_2_), could promote the migration of DCs to some extent (70–72). However, selective migration of DCs and their residence in nonlymphoid and lymphoid organs are tightly regulated events. The molecular control mechanisms need to be elucidated in future studies to lay the foundation for improving the stimulation conditions of DC vaccines in clinical trials.

## Conclusion

Personalized tumor neoantigens are highly specific to individuals, and tumor vaccines targeting neoantigens can effectively induce T cells to produce a strong immune response against tumors. However, the key to the effectiveness of personalized tumor neoantigens is that they can be efficiently taken up and processed by APCs and delivered to T cells to induce an antitumor immune response. However, the function of antigen-presenting cells in patients with malignant tumors is usually inhibited. Therefore, the treatment of patients with DC vaccines loaded with neoantigens can specifically target the tumor and ensure that DCs can exert their efficacy to the maximum extent. An increasing number of research and clinical trials are currently underway, promising to offer new hope to patients with solid tumors.

## Author Contributions

LY and LT conceptualized the study. LT and RZ finished the manuscript and figures, and XZ helped LT collect the related paper. All authors contributed to the article and approved the submitted version.

## Funding

This work was supported by the National Natural Science Foundation of China (No. 82073366), West China Hospital, Sichuan University.

## Conflict of Interest

The authors declare that the research was conducted in the absence of any commercial or financial relationships that could be construed as a potential conflict of interest.

## Publisher’s Note

All claims expressed in this article are solely those of the authors and do not necessarily represent those of their affiliated organizations, or those of the publisher, the editors and the reviewers. Any product that may be evaluated in this article, or claim that may be made by its manufacturer, is not guaranteed or endorsed by the publisher.
